# Inhalation therapy with the synthetic TIP-like peptide AP318 attenuates pulmonary inflammation in a porcine sepsis model

**DOI:** 10.1186/s12890-015-0002-6

**Published:** 2015-02-07

**Authors:** Erik K Hartmann, Alexander Ziebart, Rainer Thomas, Tanghua Liu, Arno Schad, Martha Tews, Bernd Moosmann, Jens Kamuf, Bastian Duenges, Serge C Thal, Matthias David

**Affiliations:** Department of Anesthesiology, Medical Center of the Johannes Gutenberg-University, Langenbeckstrasse 1, 55131 Mainz, Germany; Institute of Pathology, Medical Center of the Johannes Gutenberg-University, Langenbeckstrasse 1, 55131 Mainz, Germany; Institute of Pathobiochemistry, Medical Center of the Johannes, Gutenberg-University, Duesbergweg 6, 55128 Mainz, Germany

**Keywords:** Lectin-like domain, TIP peptide, AP318, Pulmonary inflammation, Sepsis, Pig model

## Abstract

**Background:**

The lectin-like domain of TNF-α can be mimicked by synthetic TIP peptides and represents an innovative pharmacologic option to treat edematous respiratory failure. TIP inhalation was shown to reduce pulmonary edema and improve gas exchange. In addition to its edema resolution effect, TIP peptides may exert some anti-inflammatory properties. The present study therefore investigates the influence of the inhaled TIP peptide AP318 on intrapulmonary inflammatory response in a porcine model of systemic sepsis.

**Methods:**

In a randomized-blinded setting lung injury was induced in 18 pigs by lipopolysaccharide-infusion and a second hit with a short period of ventilator-induced lung stress, followed by a six-hour observation period. The animals received either two inhalations with the peptide (AP318, 2×1 mg kg^−1^) or vehicle. Post-mortem pulmonary expression of inflammatory and mechanotransduction markers were determined by real-time polymerase chain reaction (IL-1β, IL-6, TNF-α, COX-2, iNOS, amphiregulin, and tenascin-c). Furthermore, regional histopathological lung injury, edema formation and systemic inflammation were quantified.

**Results:**

Despite similar systemic response to lipopolysaccharide infusion in both groups, pulmonary inflammation (IL-6, TNF-α, COX-2, tenascin-c) was significantly mitigated by AP318. Furthermore, a Western blot analysis shows a significantly lower of COX-2 protein level. The present sepsis model caused minor lung edema formation and moderate gas exchange impairment. Six hours after onset pathologic scoring showed no improvement, while gas exchange parameters and pulmonary edema formation were similar in the two groups.

**Conclusion:**

In summary, AP318 significantly attenuated intrapulmonary inflammatory response even without the presence or resolution of severe pulmonary edema in a porcine model of systemic sepsis-associated lung injury. These findings suggest an anti-inflammatory mechanism of the lectin-like domain beyond mere edema reabsorption in endotoxemic lung injury in vivo.

## Background

Sepsis is a frequent cause of acute respiratory distress syndrome (ARDS). Sepsis-induced ARDS can be caused by a primary pulmonary focus or a systemic transmission, while isolated ARDS may also trigger a systemic response leading to multiple organ failure [1,2]. A key mediator of early pulmonary inflammation is tumor-necrosis-factor-alpha (TNF-α), which is known to exert pleiotropic effects [3,4]. The lectin-like domain of TNF-α, which is located at the tip of the pyramidal molecule, elicits edema resolution and endothelial barrier sealing effects independently from the pro-inflammatory main effects of the cytokine. The lectin-like domain therefore contributes to the dichotomal role of TNF-α in severe inflammatory conditions [4]. This domain can be mimicked by synthetic TIP peptides, which recently emerged as a novel approach for treatment of ARDS and are currently tested for clinical application [4-6]. Via activation of epithelial sodium channels (ENaC) in type II alveolar cells TIP peptides help to create an osmotic gradient leading to resolution of alveolar edema [7-9]. Edema formation through microvascular hyperpermeability can also be limited by the TIP peptide [8,10]. Furthermore, inhalative application was shown to limit inflammatory response in terms of leucocyte infiltration and reactive oxygen species generation [11]. In a recent porcine model of bronchoalveolar lavage induced ARDS inhalation of the TIP peptide AP301 (APEPTICO, Vienna, Austria) was effective in improving the overall pulmonary function, which was associated with edema reduction [12]. These findings have not been confirmed in vivo under conditions of systemic sepsis or severe inflammatory response. Based on previous in vitro data the synthetic peptide AP318 may represent a more potent variant of the initially synthesized TIP peptide [13,14], but only showed comparable effects to the first TIP peptide AP301 in a porcine model in vivo [15]. To further characterize the effects of the lectin-like domain on intrapulmonary inflammation, a porcine model of lipopolysaccharide (LPS)-induced sepsis with an additional ventilator-induced lung injury (VILI) component was examined. Our primary hypothesis was that repetitive AP318 inhalation would lead to an attenuation of intrapulmonary inflammatory response, and secondary improve gas exchange and overall lung injury.

## Methods

This study was approved by the state and institutional animal care committee (Landesuntersuchungsamt Rheinland-Pfalz, Koblenz, Germany; approval number 23 177-07/G12-1-058), and conducted in accordance with institutional guidelines of the Johannes Gutenberg-University Mainz, Germany. 18 juvenile pigs (weight 25–27 kg) were examined in a randomized, investigator-blinded setting.

### Anesthesia and instrumentation

After sedation with intramuscular injection of ketamine (8 mg kg^−1^) and midazolam (0.2 mg kg^−1^) and vascular access by ear vein puncture, anesthesia was induced and maintained by intravenous propofol and fentanyl administration (8–12 mg kg^−1^ h^−1^ / 0.1-0.2 mg h^−1^). A single dose of atracurium (0.5 mg kg^−1^) was applied to facilitate orotracheal intubation. Ventilation (Respirator: AVEA®, CareFusion, USA) was started in pressure-controlled mode with a tidal volume of (V_t_) of 8 mL kg^−1^, positive end-expiratory pressure (PEEP) of 5 cmH_2_O, fraction of inspired oxygen (FiO_2_) of 0.4 and a variable respiratory rate to maintain normocapnia. A balanced saline solution (Sterofundin iso, B. Braun, Germany) was continuously infused at a rate of 10 mL kg^−1^ h^−1^. Vascular catheters were placed ultrasound-guided in Seldinger’s technique and under sterile conditions: an arterial line, a pulse contour cardiac output catheter (PiCCO, Pulsion Medical Systems, Germany) and central venous line were inserted via femoral access. An introducer for a pulmonary artery catheter was placed via the right internal jugular vein. Ventilatory and extended hemodynamic parameters were recorded continuously (Datex S/5, GE Healthcare, Germany). Body temperature was measured by a rectal probe and normothermia was maintained by body surface warming.

### Experimental protocol

**Figure 1:**
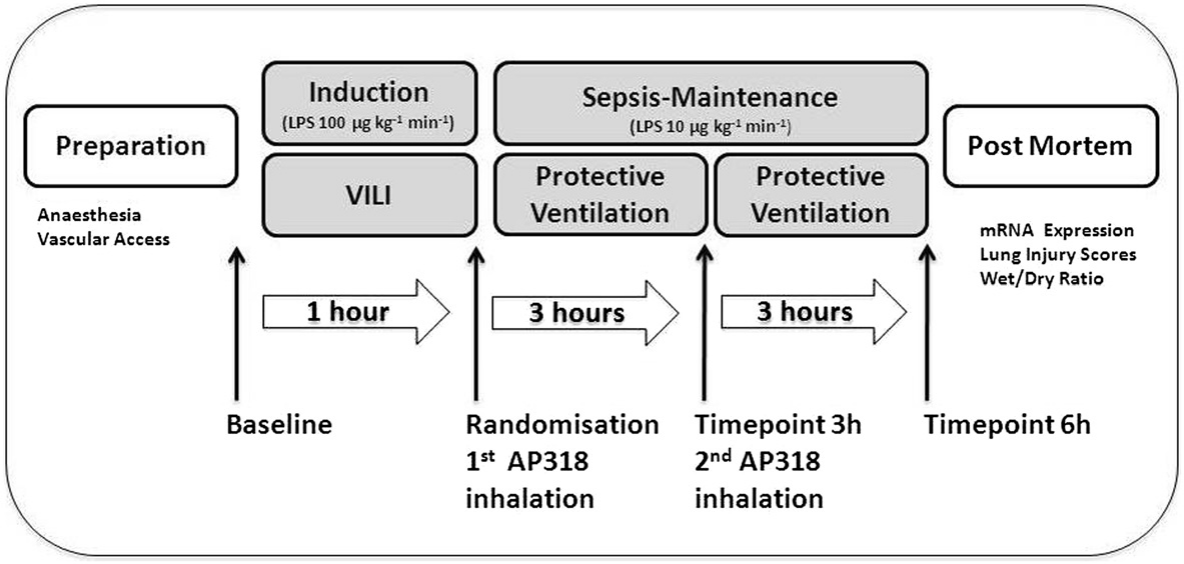
**Experimental protocol.**

Following instrumentation baseline parameters were assessed at healthy state**.** Figure 1 summarizes the experimental protocol: systemic inflammation was induced by continuous LPS infusion (Escherichia coli serotype O111:B4, Sigma-Aldrich, Switzerland) for one hour at 100 μg kg^−1^ h^−1^, followed by 10 μg kg^−1^ h^−1^ for the entire experiment. Initial high-dose infusion was combined with a non-protective ventilation setting (V_t_ 25 mL kg^−1^, zero PEEP, FiO_2_ 1.0) to add a VILI component. Afterwards the ventilation mode was switched to a more lung protective setting: V_t_ of 8 mL kg^−1^, PEEP 5 cmH_2_O, FiO_2_ of 0.4, and a variable respiratory rate to maintain a pH > 7.2. The animals were monitored over six hours after sepsis induction. During the induction phase a non-participant randomized the animals into two groups and prepared the peptide solution for blinded endotracheal inhalation:

1. AP318 group (1 mg kg^−1^ AP318 at zero and three hours, n = 9)
2. Control group (CTRL; vehicle solution at zero and three hours, n = 9)

The cognate TIP variant AP318 was provided by APEPTICO (Vienna, Austria) and delivered as lyophilisate at – 20°C. For preparation and conduction of the inhalation by means of a clinical nebulizer (Aeroneb ProX, Aerogen Ltd, Ireland) a previously established, standardized protocol was applied [15]. To maintain hemodynamic stability (mean arterial pressure > 60 mmHg) additional fluid boli were administered (150 ml of balanced saline or hydroxyethyl starch once every hour). Persisting instability was treated by continuous central venous noradrenaline infusion. At the end of the experiments the animals were killed in deep general anesthesia by intravenous injection of propofol (200 mg) and potassium chloride (40 mval).

### Gene expression analysis and immunoblotting

To determine intrapulmonary inflammation mRNA levels of pro-inflammatory cytokines interleukin-1β (IL-1β), interleukin-6 (IL-6), TNF-α, and enzymes prostaglandin G/H synthase-2 (COX-2) and inducible nitric oxide synthase (iNOS) were quantified. Amphiregulin and tenascin-c expression levels were examined as surrogates of mechanical stress and remodeling. Additionally, ENaC expression was analyzed by quantification of the channel’s β-subunit. Four representative samples from the left lung (upper/lower lobe, each dependent/non-dependent) were collected, snap frozen in liquid nitrogen and stored at −80°C. RNA extraction and quantification procedure by real-time polymerase chain reaction (Lightcycler 480 PCR System, Roche Applied Science, Germany) was conducted as previously described in detail [16-18]. mRNA expression data were normalized against peptidylprolyl isomerase A (PPIA) as control gene. The applied primer sequences are summarized in Table 1. To determine tissue protein content of COX-2, the homogenates were analyzed by Western blotting following standard protocols [19]. Lung tissue samples (~100 mg) were homogenized in 4 ml solubilization buffer [100 mM Tris–HCl, pH 7.4, 20% sucrose, 0.5% SDS, 1× protease inhibitor cocktail (P2714 from Sigma-Aldrich, USA)] at 4°C using an Ultra-Turrax dispersing device (IKA, Germany). Protein content in the obtained homogenates was determined by bicinchoninic acid protein assay (Pierce, USA). The employed polyclonal, rabbit anti-cyclooxygenase-2 antibody (1:200; ab15191 from Abcam, UK) detected a single, predominant band of ~70 kDa in these porcine samples. COX-2 signal intensity was normalized for Ponceau S staining intensity of the applied total protein between ~25 kDa and ~170 kDa. Densitometric quantification was achieved using the commercial image analysis software AIDA (Raytest, Germany).

**Table 1:** **Specific real-time PCR primers**

**PCR assay**	**Oligonucleotide sequence (5′-3′)**	**GenBank no.**
IL-1β	S: ACCCTgCAgCTggAggAT	NM_214055
	A: CCTTTggAgTTTCCCAggA	
IL-6	S: CCAATCTgggTTCAATCAggA	NM_214399
	A: gTggTggCTTTgTCTggATTC	
	FL: TgTCgAggCTgTgCAgATTAgTACCA(--FL)	
	CY5: (L670-)gCACTgATCCAgACCCTgAggCAA(--PH)	
TNF-α	S: CCCAgAAggAAgAgTTTCCA	NM_214022
	A: CggCTTTgACATTggCTACA	
	FL: ggCCCAAggACTCAgATCATCgTC(--FL)	
	CY5: (L670-)CAAACCTCAgATAAgCCCgTCgC(--PH)	
iNOS	S: gATggCACCATCATAggggAC	NM_001143690
	A: ggCACCCTgggAACTCAA	
	FL: TGGAACACCCCAAATACGAGTGGTTCC(--FL)	
	CY5: (L670-)GGAGCTGGAGCTGAAGTGGTACGCCC(--PH)	
COX-2	S: CCCCTTCTgCCTgACgC	NM_214321
	A: CTCTgCTCTggTCgATTgAgg	
	FL: TCTATCTTACTggAACATggCATCACCCA(--FL)	
	CY5: (L670-)TTTgTTgAATCATTTAgCAggCAAATTgCT(--PH)	
AREG	S: ATTATGCTGCTGGACTGGAC	NM_214376
	A: TCGCTACCAGAAGGCATTT	
TNC	S: GAGACCTGACTGCTACAGAG	NM_214230
	A: CACAACGACTTCCTTGAGTG	
PPIA	S: CTTTCACAgAATAATTCCAggATT	NM_214353
	A: ggACAAgATgCCAggACC	
	FL. ATgCTTCAggATAAAATTCTCATCATCAAA(--FL)	
	CY5: (L670-)TTCTCTCCATAgATggACTTgCCACCA(--PH)	
sENACg	S: ACTTCACCCCCATCTTCCAC	XM_003357614
	A: TTCAGGCCAAACTCAGCTC	

S: sense primer, A: anti-sense primer, FL: fluorescein, CY5: CY5-labelled. IL-1β, Sus scrofa interleukin 1 beta (IL1B), mRNA; IL-6, Sus scrofa interleukin 6 (interferon, beta 2) (IL6), mRNA; TNF-α, Sus scrofa tumor necrosis factor (TNF superfamily, member 2) (TNF), mRNA; iNOS, Sus scrofa nitric oxide synthase 2, inducible (NOS2), mRNA. COX-2, Sus scrofa prostaglandin G/H synthase-2 (PGHS-2), mRNA; AREG, Sus scrofa amphiregulin (AREG), mRNA; TNC, Sus scrofa tenascin C (TNC), mRNA; PPIA, Sus scrofa peptidylprolyl isomerase A (cyclophilin A) (PPIA), mRNA; sENACg, Sus scrofa sodium channel, non-voltage-gated 1, beta subunit (SCNN1B), transcript variant X1, mRNA.

### Hematological parameters

Blood gas values were obtained using a Rapidlab 248 device (Bayer Healthcare, Germany). Hematological parameters were determined during baseline, at the end of sepsis/VILI induction, and after three and six hours. Lactate plasma levels, leucocyte and platelet counts were analyzed by the Institute of Laboratory Medicine, Medical Center of the Johannes Gutenberg-University. The plasma levels of IL-6 and TNF-α were determined by quantifying enzyme linked immunosorbent assays (Porcine IL-6 Quantikine ELISA, Porcine TNF-a Quantikine ELISA, R&D Systems, Germany) according to the manufacturer’s instructions.

### Histopathological and lung water content assessment

The lungs were removed en-bloc after thoracotomy and a macroscopic lung injury score [15] was applied: four ventral and dorsal segments (each upper/lower right, upper/lower left) of the lung surface were examined for hemorrhage and congestion (2 points > 50%, 1 point for < 50%, 0 points for no or minimal changes). The left lung was weighted immediately after removal and dried afterwards at 60°C for 72 hours to determine the dry weight and wet to dry ratio. From the right lung samples of the bronchoalveolar lavage fluid were taken and snap frozen in liquid nitrogen to determine the alveolar protein content and cytokine levels by ELISA. The right lung was then fixed in 10% buffered formalin. Representative tissue samples were paraffin embedded and cut for hematoxylin-eosin staining. A blinded investigator under supervision of a senior pathologist performed the histopathological assessment. In different lung regions (non-dependent periphery and bronchial, dependent periphery and bronchial) morphological changes were rated for seven criteria (alveolar edema, interstitial edema, hemorrhage, inflammatory infiltration, epithelial damage, microatelectasis and overdistension). The severity of each parameter ranged from 0 (no occurrence) to 5 points (complete field). For every lung region we used the mean value of four non-overlapping fields of view. The sum of the regional scores in all lung regions adds to a maximum injury score of 140 points (7 parameters × 5 maximum points per parameter from 4 lung regions). Additionally, we assessed the regional distribution of each parameter by comparison of the dependent versus non-dependent lung regions. Similar scoring procedures were described previously [20,21].

### Statistical analysis

**Figure 2:**
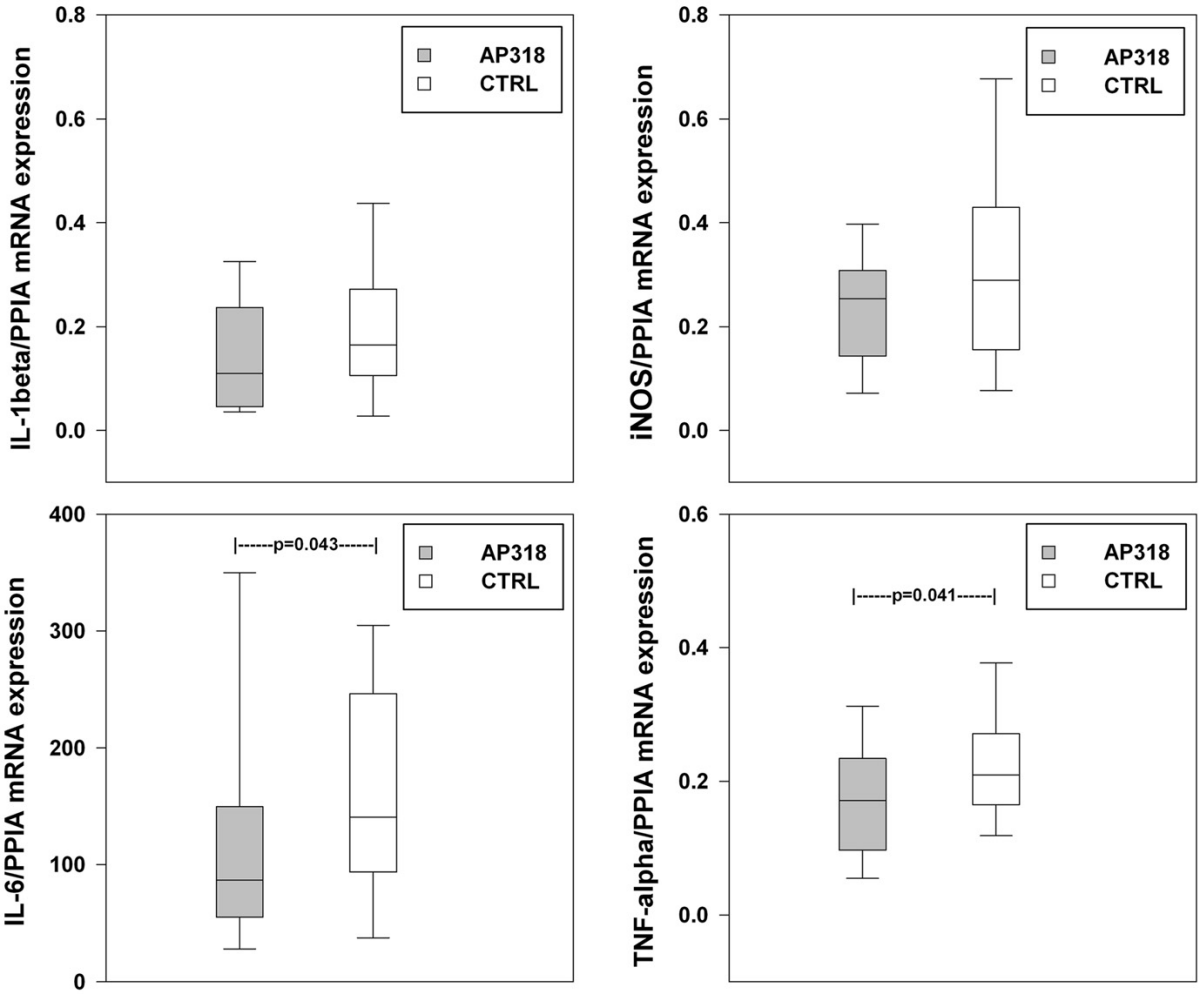
**Pulmonary expression of inflammatory cytokines and enzymes.** Intrapulmonary mRNA expression [mRNA copies] of inflammatory cytokines normalized to peptidylprolyl isomerase A (PPIA): IL-1β, IL-6, TNF-α, and iNOS. Significant intergroup differences indicated by P values.

**Figure 3:**
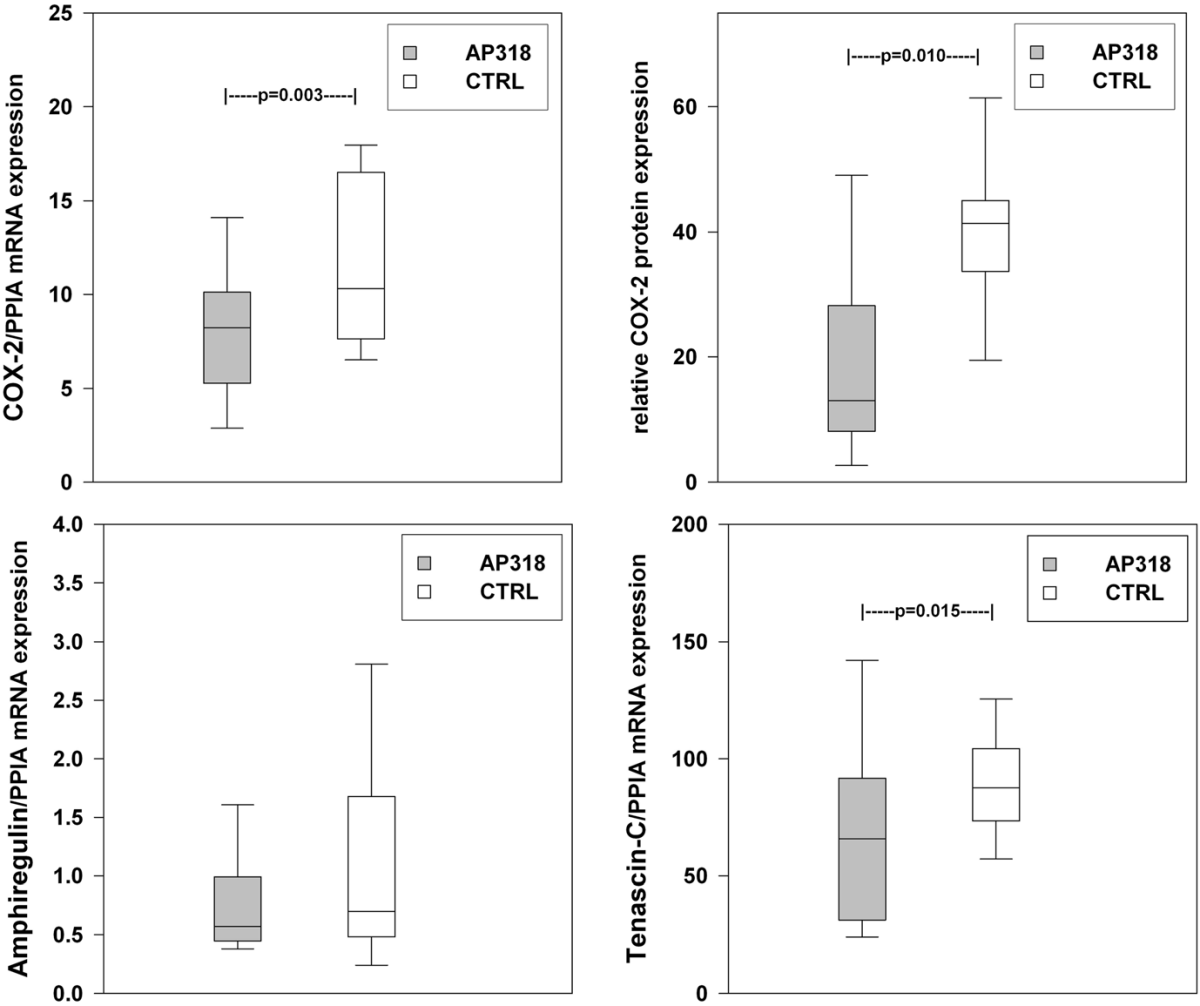
**COX-2 and mechanotransduction.** Intrapulmonary mRNA expression [mRNA copies] of COX-2, amphiregulin and tenascin-c normalized to peptidylprolyl isomerase A (PPIA). COX-2 protein expression by Western Blot analysis. Significant intergroup differences indicated by P values.

**Figure 4:**
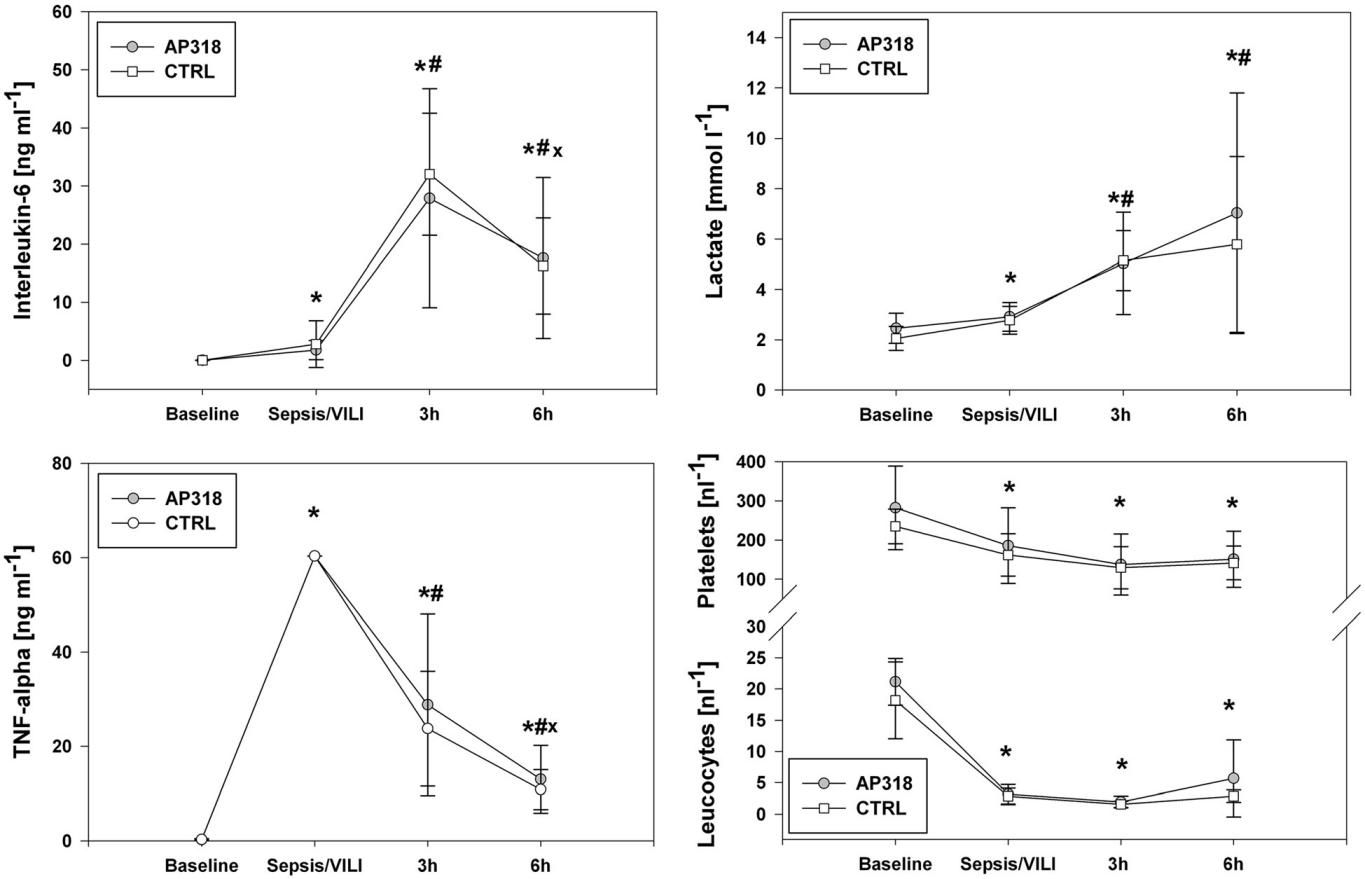
**Systemic hematological parameters and inflammatory cytokines.** Time courses of systemic TNF-α, IL-6, lactate, leucocytes, and platelets. *Indicates P < 0.05 vs. baseline value, # P < 0.05 vs. Sepsis/VILI and × P < 0.05 vs. 3 h value. No significant intergroup differences. Sepsis/VILI values of TNF-α exceed the detection capacities of the available assays.

**Figure 5:**
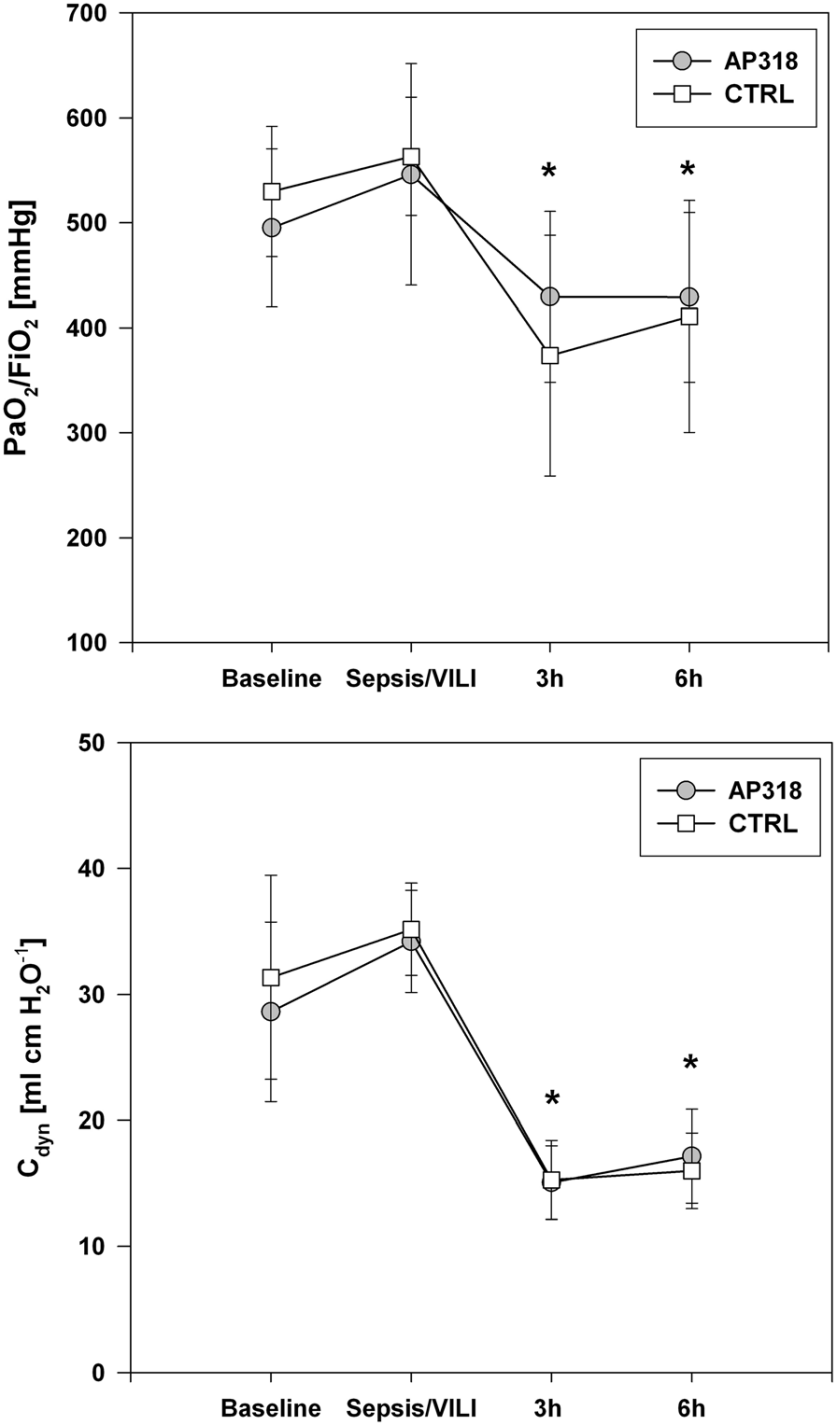
**Gas exchange and respiratory mechanics.** Time courses of PaO_2_/FiO_2_ and C_dyn_. *P < 0.05 vs. Baseline. No intergroup differences.

**Figure 6:**
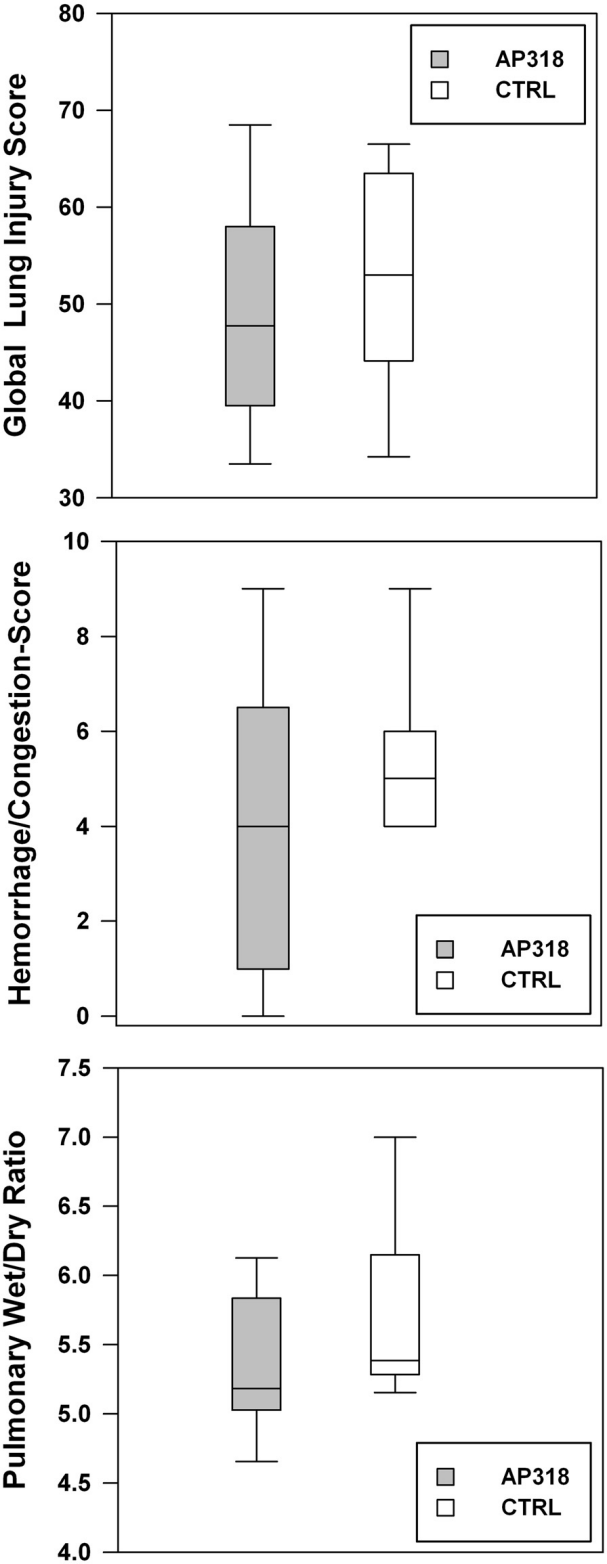
**Pathological assessment of the lungs.** Global histopathological respectively macroscopic lung injury scoring and pulmonary wet to dry ratio. No significant intergroup differences.

Data are expressed as mean ± standard deviation (SD) or box-plots. For intergroup comparisons of the primary parameters (pulmonary mRNA expression, Figures 2 and 3) the Mann–Whitney-U-Test was used. Intragroup time courses of repetitively measured parameters were analyzed by Friedman ANOVA on ranks and post-hoc SNK-Test. Differences of secondary parameters (Figures 4, 5 and 6) between the two groups were assessed by the Mann–Whitney-U-Test. If multiple testing was performed, P values were adjusted by the Bonferroni method. P values below 0.05 were regarded as significant. Comparisons of physiological data for each time point (Table 1) were analyzed in an explorative manner by one-way ANOVA. The statistical software SigmaPlot 12.5 (Systat Inc., USA) was used.

## Results

### Pulmonary and systemic inflammatory markers

Intrapulmonary mRNA quantification yielded significantly lower overall expression of COX-2, TNF-α and IL-6 following AP318 inhalation, while the differences in IL-1β and iNOS expressions failed to reach significance (Figures 2 and 3). Additionally, a Western blot shows a significantly decreased COX-2 protein level in the lung tissue (Figure 3). ENaC expression was comparable in both groups (AP318 17.5 ± 9.2, CTRL 13.6 ± 7.4 [mRNA copies]; P = 0.12). Furthermore a decreased tenascin-c expression was detected after AP318 inhalation. No relevant locoregional variations were present. IL-6 and TNF-α levels from the bronchoalveolar lavage fluid were not different. Secondary, the systemic inflammatory response did not differ between the two groups. LPS infusion led to a sustained and persisting leucopenia. This was accompanied by decreases in platelet count and rising lactate levels. Plasma levels of IL-6 and TNF-α increased significantly in both groups with a peak within three hours (Figure 4).

### Physiological data

Table 2 summarizes the time charts of hemodynamic and respiratory parameters. After sepsis/VILI induction the quotient of arterial partial pressure of oxygen and FiO_2_ (PaO_2_/FiO_2_) did not decrease. Afterwards PaO_2_/FiO_2_ and dynamic lung compliance (C_dyn_) significantly decreased within three hours in both groups and persisted without recovery (Figure 5). The two groups showed no significant differences. Hemodynamic parameters were stable during baseline and sepsis/VILI induction, while over six hours continuous noradrenaline infusion was required in similar dosages (Table 1; AP318 8/9 animals, CTRL 7/9 animals). Hydroxyethyl starch was applied in all animals in comparable dosages.

**Table 2:** **Ventilation and hemodynamic data**

	**AP318**				**CTRL**			
**Parameter**	**Baseline**	**Sepsis /VILI**	**3 h**	**6 h**	**Baseline**	**Sepsis /VILI**	**3 h**	**6 h**
**Ventilation**								
**V**_**t**_ **(mL kg** ^**−1**^ **)**	8.8 ± 1.4	25.4 ± 2.0	8.7 ± 0.6	8.7 ± 0.7	8.6 ± 0.9	25.8 ± 0.6	8.5 ± 0.4	8.2 ± 0.6
**P**_**endinsp**_ **(cmH**_**2**_ **O)**	14 ± 2	21 ± 3	21 ± 2	19 ± 4	14 ± 1	21 ± 2	21 ± 3	19 ± 3
**RR (min** ^**−1**^ **)**	28 ± 8	9 ± 2	33 ± 7	31 ± 5	33 ± 6	9 ± 1	36 ± 6	33 ± 9
**PEEP (cmH**_**2**_ **O)**	6 ± 1	1 ± 0	5 ± 1	5 ± 1	6 ± 1	1 ± 0	5 ± 0	6 ± 2
**FiO**_**2**_	0.4	1.0	0.4	0.4	0.4	1.0	0.4	0.4
**I:E**	1:2	1:2	1:2	1:2	1:2	1:2	1:2	1:2
**PaCO**_**2**_ **(mmHg)**	43 ± 4	35 ± 3	44 ± 5	42 ± 5	45 ± 3	36 ± 5	43 ± 5	42 ± 7
**pH**	7.41 ± 0.03	7.50 ± 0.08	7.33 ± 0.08	7.34 ± 0.08	7.41 ± 0.04	7.47 ± 0.07	7.34 ± 0.05	7.32 ± 0.08
**Hemodynamics**								
**MAP (mmHg)**	93 ± 13	103 ± 11	66 ± 13	68 ± 8	99 ± 13	115 ± 12	69 ± 12	62 ± 12
**CO (L min** ^**−1**^ **)**	4.6 ± 0.7	4.7 ± 0.7	3.2 ± 0.9	3.9 ± 1.2	4.5 ± 0.5	4.6 ± 0.7	3.2 ± 0.5	3.4 ± 0.7
**CVP (mmHg)**	12 ± 3	11 ± 2	12 ± 3	13 ± 2	11 ± 3	11 ± 3	13 ± 3	13 ± 3
**MPAP (mmHg)**	22 ± 3	23 ± 3	33 ± 5	30 ± 8	21 ± 5	26 ± 4	37 ± 8	33 ± 12
**NA (μg kg** ^**−1**^ **min** ^**−1**^ **)**	0	0	0.8 ± 1.3	2.7 ± 5.0	0	0	0.3 ± 0.5	3.1 ± 5.7

Data are presented as mean ± SD, no relevant intergroup differences. V_t_: tidal volume; P_endinsp_: end-inspiratory pressure; PEEP: positive end-expiratory pressure; RR: respiratory rate; FiO_2_: fraction of inspired oxygen; I:E: inspiration to expiration quotient; PaCO_2_: arterial partial pressure of carbon dioxide; MAP: mean arterial pressure; CO: cardiac output; CVP: central venous pressure; MPAP: mean pulmonary arterial pressure; NA: noradrenaline dosage.

### Pathologic parameters

Post-mortem macroscopic and histologic evaluations yielded the presence of a sustained lung injury in both groups. The AP318 group shows lower scoring values, but fails to reach significance (Figure 6). The alveolar protein content was minimal in both groups. The most pronounced features of the histopathological scoring were inflammatory infiltration, overdistension, and atelectasis with edema formation playing a minor role. The CTRL group featured a higher grade of hemorrhage (Table 3). No relevant differences were detected regarding the ventral to dorsal distribution.

**Table 3:** **Distribution of histopathological lung injury summarized in figure** **3**

	**non-dependent**		**dependent**	
**Parameter**	**AP318**	**CTRL**	**AP318**	**CTRL**
**Alveolar edema**	0 ± 0	0.2 ± 0.6	0 ± 0	0.1 ± 0.2
**Interstitial edema**	1.0 ± 0.7	1.2 ± 1.1	1.0 ± 0.5	0.9 ± 0.7
**Hemorrhage**	0.3 ± 0.6	1.3 ± 1.2*	0.5 ± 0.8	1.2 ± 1.1*
**Inflammatory infiltration**	3.7 ± 0.9	3.5 ± 0.8	3.6 ± 1.0	3.7 ± 0.8
**Epithelial destruction**	0 ± 0	0.4 ± 0.9	0.1 ± 0.3	0.1 ± 0.2
**Microatelectasis**	3.6 ± 1.1	3.9 ± 1.2	3.3 ± 1.4	3.4 ± 1.3
**Overdistension**	3.9 ± 0.8	3.6 ± 1.1	3.5 ± 1.0	3.5 ± 0.9

Data of lung regions (each containing periphery and bronchial area) are expressed as mean ± SD.

*Indicates P < 0.05 vs. AP318 group.

## Discussion

The key results of the present porcine model of LPS/VILI-induced lung injury are: (1) AP318 significantly mitigates pulmonary mRNA expression of inflammatory mediators despite comparable systemic response to LPS exposition and (2) gas exchange or histopathological injury did not improve in the presence of low-grade pulmonary edema within six hours post insult.

### Model characteristics

LPS is exposed as glycolipids of gram-negative bacteria in systemic bacteremia and can trigger inflammatory response to the point of septic shock and cardio-circulatory failure. Systemic effects of LPS in pigs include hemodynamic deterioration along with increased pulmonary arterial pressure and acute leucopenia [22], which is consistent with our findings. Intrapulmonary changes due to LPS infusion include accumulation of leucocytes and alveolar macrophages, as well as edema formation and endothelial injury [23]. In contrast to other models (i.e. bronchoalveolar lavage), no immediate atelectases and gas exchange impairment are generated [23]. In pigs LPS-induced lung changes measured by computer tomographic imaging and histopathologic scoring can develop over several hours without fulfillment of ARDS criteria [24]. Endotoxemic shock and therapy-refractory hemodynamic failure limit the maximum possible LPS infusion dosages in experimental models.

Regarding our primary hypothesis we designed this model to focus inflammatory response within the lung and not extensive edema formation or severe gas exchange impairment. The latter aspects were already assessed in our prior studies [12,15]. The present model causes a pulmonary lesion with a pronounced inflammatory response, which presents as significant worsening of PaO_2_/FiO_2_, respiratory mechanics, and post-mortem lung injury. Nevertheless, the complete pattern of ARDS according to the current criteria is not achieved despite addition of one-hour high V_t_ ventilation. This kind of short-term VILI does not induce sustained lung injury in healthy pigs [25]. However, synergistic effects of LPS infusion and VILI are used to achieve a full pattern human-like ARDS in experimental models [22].

### Influence on inflammatory response

In response to LPS exposure TNF-α and IL-1β are released into the systemic circulation. In early ARDS alveolar macrophages are the main source of inflammatory cytokines that trigger inflammatory response by e.g. enhancing neutrophil accumulation [26,27]. We found high circulating plasma cytokine levels, which in comparison to their baselines values showed peaks immediately after sepsis/VILI induction (TNF-α) or three hours following induction (IL-6). Pathophysiological relevance is supported by data demonstrating that early and high circulating levels of IL-6 are associated with increased mortality [9]. Due to the strict local application of AP318 and ongoing LPS infusion a systemic effect appears unlikely, which is supported by our plasma cytokine levels. Interestingly, repetitive AP318 inhalation significantly attenuated pulmonary expression of several key inflammatory markers. Western blot analysis of the exemplarily chosen marker COX-2 demonstrated a significantly reduced content also on the protein level, indicating that the duration of the experiment (6 h) was sufficient to affect not only transcription, but also and in parallel direction translation. This observation authenticates the general use of real-time polymerase chain reaction to characterize the effects of AP318 on multiple markers in the described model.

The TIP peptide’s impact on lung inflammation was investigated previously in experimental ischemia and reperfusion–related lung injury, which demonstrated reduced neutrophil accumulation and reactive oxygen species generation, but failed to show differences in cytokine levels [11]. In contrast to the present study, the latter quantified cytokine levels in bronchoalveolar lavage fluid, whereas we also determined inflammatory marker genes directly in lung tissue. Similar to these data, we found no differences in cytokine levels from the lavage fluid. Low level of edema formation in our study may have resulted in a limited effect (Figure 6, Table 3) on the alveolar cytokine levels. The level of expression is not dependent on the localization within the lung, which can be attributed to the systemic character of LPS infusion and the ventilation mode following the one-hour induction phase. Lung protective ventilation is known to avoid cyclical recruitment [28], whereas on-going VILI leads to regional differences in extend of inflammatory response [24]. The underlying molecular mechanisms of TIP have not been fully elucidated [11], and the present study was not design to clarify them. TIP directly activates ENaC by binding upon the channel’s α-subunit [29]. In the presence of LPS ENaC expression shows a biphasic curve with an upregulation within eight hours followed by a downregulation [30]. ENaC quantification yielded no significant differences between the two groups, which assumes that AP318 does not increase ENaC expression in the present model. Improvement of gas exchange and reduction of neutrophil invasion by TIP are linked, as both are inhibited by pharmacological ENaC blockade [11]. Our data, however, for the first time demonstrate an attenuation of pulmonary inflammatory response without significant edema reduction or improved gas exchange. Hence, edema absorption itself is hardly responsible for mitigation of inflammatory response despite being linked to ENaC. Furthermore, protein kinase C-α activation, which is induced upon Ca^2+^ influx by bacterial cytolysins like pneumolysin or listeriolysin, is inhibited by TIP [10,31]. This reduces permeability and reactive oxygen species release. Further studies, however, need to clarify, if mitigating of inflammatory response by a TIP peptide is a primary mechanism or occurs secondary to the mentioned mechanisms.

In order to differentiate between LPS and VILI-induced lung injury, we quantified markers for mechanical and inflammatory stress in amphiregulin and tenascin-c. Amphiregulin mRNA levels are induced exclusively following VILI, but not by LPS administration [25]. Tenascin-c, an extracellular matrix glycoprotein, is particularly involved in early inflammation and induced by inflammatory cytokines, lung remodeling, and fibroproliferation [32,33]. Tissue amphiregulin levels were not different between AP318 and CTRL group, indicating that mechanical stress was similar between both groups and that AP318 did not protect the lung from mechanical stress. Tenascin-c on the other hand was significantly lower in the AP318 group, suggesting that AP318 inhalation mitigates the activity associated with inflammation.

### Influence on pulmonary function

In the present study gas exchange impairment and respiratory mechanics were not improved by AP318 inhalation. This is in contrast to previous data reporting a rapid [7,12,15] and lasting [11] amelioration of gas exchange or respiratory mechanics impairment after TIP peptide inhalation. The failure of compensatory mechanisms may contribute to the low efficacy of AP318 to improve gas exchange despite mitigation of inflammation: LPS significantly impairs pulmonary perfusion and inhibits hypoxic pulmonary vasoconstriction [34] and LPS-induced lung injury respond poorly to lung recruitment [35]. Recent data suggest that lung water content closely correlates with the ventilation/perfusion-distribution in non-septic experimental ARDS [36]. Pulmonary edema was not reduced by AP318, although the main effect of the TIP peptides is stimulation of ENaC and consequently edema resolution [9,14]. A porcine ARDS model of bronchoalveolar lavage and flooding as well as an ex vivo exo-/endotoxin rabbit model demonstrated that TIP inhalation rapidly resolved a preformed edema [8,12]. In contrast to these publications the present model was intentionally not accompanied with a relevant alveolar edema. Different model characteristics may therefore account for the lack of efficacy. Hence, the present data suggest that amelioration of gas exchange by the lectin-like domain appears to be linked rather to edema resolution and corresponding lung recruitment than to anti-inflammatory properties.

### Implications and limitations of AP318 inhalation

Three key mechanisms need to be addressed to allow recovery from ARDS: resolution of the pulmonary edema, stabilization of the injured epithelial and endothelial structures and attenuation of the inflammatory response [1]. Therapeutic concepts primarily emphasize a strict adherence to lung protective ventilation and, if applicable, treatment of the underlying disease. Experimental data indicate that TIP peptide inhalation may target at least two of these pathomechanisms by stimulation of alveolar edema reabsorption and reversal of microvascular hyperpermeability [8,9,12]. The complex pattern of ARDS requires therapeutic strategies, which address multiple pathways. TIP peptides are therefore promising drugs to address endothelial barrier integrity, edema formation and inflammation simultaneously. Via inhalation the effects are locally restricted making systemic effects less likely [5,11]. Severe alveolar epithelial injury on the other hand may also limit beneficial pulmonary effects [37]. Hence, the lectin-like domain may be better suited for early states of edematous respiratory failure or ARDS rather than in severe or late fibroproliferative phases.

## Conclusion

In a porcine model of systemic inflammatory response related lung injury with short-term VILI a repetitive inhalation of AP318 significantly attenuated the intrapulmonary expression of inflammatory marker genes. These findings provide new insights into the mechanisms of the TNF-α’s lectin-like domain beyond mere edema reduction and suggest for the first time an in vivo anti-inflammatory effect in endotoxemic lung injury.

## Abbreviations

ARDS: acute respiratory distress syndrome
C_dyn_: dynamic compliance
COX-2: prostaglandin G/H synthase-2
CTRL: control group
ENaC: epithelial sodium channels
FiO_2_: fraction of inspired oxygen
IL-1β: interleukin-1β
IL-6: interleukin-6
iNOS: inducible nitric oxide synthase
LPS: lipopolysaccharide
PaO_2_: arterial partial pressure of oxygen
PEEP: positive end-expiratory pressure
PPIA: peptidylprolyl isomerase A
SD: standard deviation
TIP: synthetic peptide mimicking the lectin-like domain of TNF-α
TNF-α: tumor-necrosis-factor-α
VILI: ventilator-induced lung injury
V_t_: tidal volume
